# Intra-medullary tuberculoma of the spinal cord presenting with typhoid and paraplegia: a case report

**DOI:** 10.1186/1752-1947-6-388

**Published:** 2012-11-13

**Authors:** Sanaullah Bashir, Akhtar Amin Memon, Maryam Sanaullah, Yasmeen Hasan

**Affiliations:** 1 Dow Medical College, Dow University of Health Sciences, Karachi, Pakistan; 2 Department of Neurology, Civil Hospital Karachi, Dow University of Health Sciences, Karachi, Pakistan

**Keywords:** Intra-medullary tuberculoma, Spinal tuberculoma, Muscle weakness

## Abstract

**Introduction:**

Intra-medullary spinal tuberculoma is a rare form of tuberculosis, with an incidence of only two in 100,000 patients with tuberculosis. We present a case of intra-medullary tuberculoma from Pakistan, which was diagnosed by radiological findings and analysis of cerebrospinal fluid using polymerase chain reaction testing.

**Case presentation:**

We present the case of a 28-year-old Sindhi male with intra-medullary tuberculoma of the spinal cord at the C3 level. Our patient was treated solely with anti-tubercular drug therapy with no surgical intervention.

**Conclusions:**

We discuss the possible clinical management of such rare cases, considering both chemotherapeutic and surgical options. Additionally, diagnostic procedures and findings are discussed; we suggest cerebrospinal fluid analysis via polymerase chain reaction and gadolinium-diethylenetriamine pentaacetic acid magnetic resonance imaging as important chemical and radiological tests to be performed in such cases.

## Introduction

Tuberculosis continues to be an important health concern in developing countries such as Pakistan, although various treatment therapies have been devised. Pakistan stands sixth among the countries with the highest prevalence rate of tuberculosis, and makes up 43 percent of the disease burden in the Eastern Mediterranean Region [1].

Intra-medullary spinal tuberculoma is a rare form of tuberculosis, with an incidence of only two cases in every 100,000 patients with tuberculosis [2]. Cascino and Dibble reported the first case of intra-medullary tuberculoma of the spinal cord in 1956 [3]. MacDonell *et al*. reviewed a total of 18 cases of intra-medullary tuberculomas of the spinal cord reported over a period of 30 years [4]. Ramdurg *et al*. reported a series of 15 cases over a period of 21 years [5]. It develops either due to hematogenous dissemination from a primary foci, or spinal fluid infection. The ratio of intra-cerebral to intra-spinal tuberculomas is reported to be 42:1 [6].

We present a case of intra-medullary tuberculoma from Pakistan, which was diagnosed by radiological findings and analysis of cerebrospinal fluid using polymerase chain reaction (PCR) testing. It was cured solely by anti-tubercular drug therapy.

## Case presentation

A 28-year-old Sindhi man male presented to our facility with a history of typhoid fever and flaccid paraplegia, anorexia, weight loss and somnolence. He developed fever four months ago, which was continuous, low grade and gradual in onset, with a non-specific pattern with bouts but without rigors and chills. It was associated with generalized fatigue and generalized body pains. He was diagnosed as having typhoid fever. The fever continued for three months and then subsided after treatment with the second-generation fluoroquinolone ciprofloxacin. Subsequently (one week later) our patient developed weakness of the left upper limb, which was gradual in onset. It was progressive and initiated distally. Over a period of four weeks, the weakness developed in a similar pattern in the remaining limbs, sufficient to inhibit our patient’s routine activities including walking, changing his clothes, buttoning clothing and combing his hair. Our patient also had pain and numbness in the hands and arms, with mild pain in the neck. There was no history of urinary or fecal incontinence. Our patient was of low socioeconomic status and his familial history was insignificant.

Systemic examination was unremarkable. Additionally, the results of a general physical examination were normal. Further, neurological examination was normal, with all the cranial nerves intact. A motor examination revealed wasting of the right thenar and hypothenar muscles and decreased tone in all four lower limbs, power 4/5 right upper limb, 4/5 left upper limb, 4/5 in right lower limb, 5/5 left lower limb, brisk reflexes, a positive Hoffman result, and bilateral plantars upgoing. Sensory, cerebellar and spinal examination results were normal, and there were no signs of meningeal irritation.

**Figure 1:**
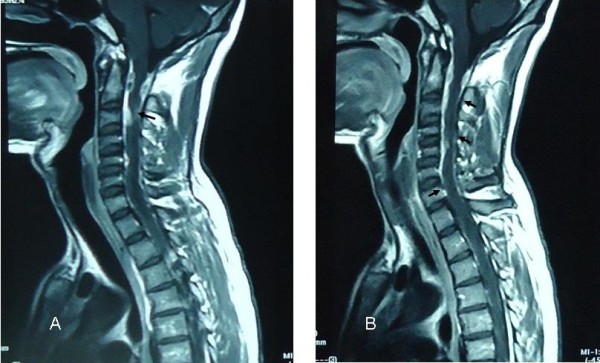
**T1-weighted magnetic resonance imaging revealing an intra-medullary tuberculoma.** Sagittal sections; multiple hyper-intense lesions are visible at C1 **(A)** (arrow) and **(B)** (top arrow), C2 and C6 levels **(B)** (bottom two arrows).

**Figure 2:**
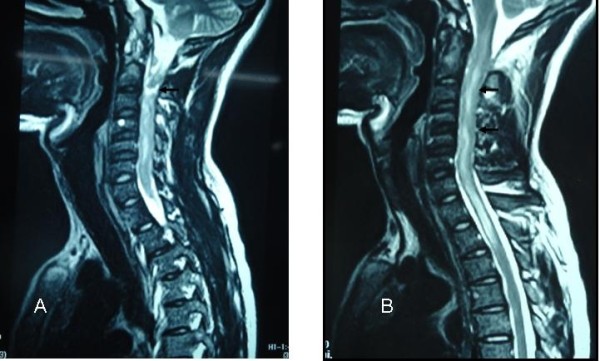
**T2-weighted magnetic resonance imaging revealing intra-medullary tuberculoma.** Sagittal sections; a hyper-intense lesion is visible along the whole cervical region **(A,B)** (arrows) and upper thoracic region **(B)**.

Magnetic resonance imaging (MRI) findings showed an intra-medullary, intra-spinal, nodular, circumferential hypo-intense enhancement, seen in the cervical region. This was more marked at the C3 level, associated with compressive effacement of the spinal cord at that point (Figure 1). The cervical cord was swollen with T2 hyper-intensity secondary to edema (Figure 2).

Differential diagnoses included cervical myelitis, multiple sclerosis, spinal tuberculosis and space occupying lesions (SOL).

The leucocyte count in his cerebrospinal fluid (CSF) was 595 cells per mm^3^, erythrocyte count 5 to 7 cells per high-power field (HPF), protein 2440mg**/**dL (normal range 20 to 45) and glucose was 29mg**/**dL (normal range 45 to 65). Results of an investigation of his CSF for Gram-staining bacteria, acid-fast bacilli (AFB) and the results of a mycological examination were all negative. A CSF sample was sent for polymerase chain reaction (PCR) testing for *Mycobacterium tuberculosis*, which gave a positive result. On the basis of all the above-mentioned findings, the diagnosis was confirmed to be acute tubercular myelitis. Our patient was given anti-tubercular drug therapy and responded remarkably well, with no surgical intervention required.

Our patient recovered fully despite the progressive nature of the disease, and he has not had any signs of paraparesis or relapse of the disease.

## Discussion

Here, we present a rare case of intra-medullary tuberculoma of the spinal cord. The symptom of progressive lower limb weakness reported by our patient was similar to that reported by MacDonnell *et al*. in 94 percent of their review of 18 cases. However, the urinary and fecal incontinence usually present in such cases was not seen in our patient.

Interestingly, the age of our patient (28 years old) coincides with the mean risk age as given by MacDonnell *et al*., and is also near to the risk age of 31 years as reported by Ramdurg *et al*. [5]. Therefore, the age group from the late 20s to early 30s can be regarded as more frequently associated with this condition. Further, women are more often affected in such cases [4], while our patient was a male.

MRI scans, particularly gadolinium-diethylenetriamine pentaacetic acid (Gd-DTPA) MRI, have been reported to be important not only for the detection of such tuberculomas, but also in the determination of induction therapies [4]. Hypo-intense ring enhancement, with or without T2 hyper-intensity, has been regarded as the characteristic MRI imaging for intra-medullary tuberculomas [7], which is similar to our findings. This further corroborates our diagnosis.

A literature review showed that lesions were found in the thoracic cord in the majority of cases [4,5,8]. Although rare, cases in the cervical region have also been reported [9]. Lin *et al*. reported a case from China where the lesion was found to be at the C3 level of the cord [9], which is the same position as the lesion in our patient.

The results of staining CSF for acid-fast bacilli were negative, while the CSF-PCR findings were positive for *Mycobacterium tuberculosis.* A similar situation was also reported by Tahir *et al*. in a case report from India [10]. Thus, we propose that CSF staining cannot be regarded as a standard diagnostic test for intra-medullary tuberculoma. In all suspected cases, negative CSF stains should be followed by CSF-PCR testing before reaching any final diagnosis.

## Conclusions

Surgery resection followed by treatment with anti-tubercular drugs is the most practiced therapy for such cases. Ramdurg *et al*. showed that out of 15 cases, 12 underwent surgery [5]. Also, the patient reported on by Lin *et al*., with a lesion at a similar level as in our patient, underwent a laminectomy [9]; our patient was given (and cured by using) only anti-tubercular drug therapies.

Therefore, via an extensive review of past cases, it has to be determined as to whether surgical resection can be kept as a secondary therapy and if priority can be given to chemotherapeutic measures while dealing with cases of intra-medullary tuberculoma of the spinal cord.

## Consent

Written informed consent was obtained from the patient for publication of this manuscript and any accompanying images. A copy of the written consent is available for review by the Editor-in-Chief of this journal.

## Competing interests

The authors declare that they have no competing interests.

## Authors’ contributions

BS took our patient’s history, performed examinations and prescribed treatment for our patient; MAA helped with the writing of the discussion; SM helped with the drafting of the case report and writing of the introduction and case presentation; HY supervised, cross-checked the findings and critically reviewed the manuscript. All authors read and approved the final manuscript.
